# Parvalbumin Interneuron Dysfunction in Neurological Disorders: Focus on Epilepsy and Alzheimer’s Disease

**DOI:** 10.3390/ijms25105549

**Published:** 2024-05-19

**Authors:** Beulah Leitch

**Affiliations:** Department of Anatomy, School of Biomedical Sciences, Brain Health Research Centre, University of Otago, Dunedin 9016, New Zealand; beulah.leitch@otago.ac.nz

**Keywords:** parvalbumin interneurons, neurological disorders, epilepsy, Alzheimer’s disease, seizures, cognitive deficits, optogenetics, chemogenetics, designer receptor exclusively activated by Designer Drugs (DREADDs)

## Abstract

Parvalbumin expressing (PV+) GABAergic interneurons are fast spiking neurons that provide powerful but relatively short-lived inhibition to principal excitatory cells in the brain. They play a vital role in feedforward and feedback synaptic inhibition, preventing run away excitation in neural networks. Hence, their dysfunction can lead to hyperexcitability and increased susceptibility to seizures. PV+ interneurons are also key players in generating gamma oscillations, which are synchronized neural oscillations associated with various cognitive functions. PV+ interneuron are particularly vulnerable to aging and their degeneration has been associated with cognitive decline and memory impairment in dementia and Alzheimer’s disease (AD). Overall, dysfunction of PV+ interneurons disrupts the normal excitatory/inhibitory balance within specific neurocircuits in the brain and thus has been linked to a wide range of neurodevelopmental and neuropsychiatric disorders. This review focuses on the role of dysfunctional PV+ inhibitory interneurons in the generation of epileptic seizures and cognitive impairment and their potential as targets in the design of future therapeutic strategies to treat these disorders. Recent research using cutting-edge optogenetic and chemogenetic technologies has demonstrated that they can be selectively manipulated to control seizures and restore the balance of neural activity in the brains of animal models. This suggests that PV+ interneurons could be important targets in developing future treatments for patients with epilepsy and comorbid disorders, such as AD, where seizures and cognitive decline are directly linked to specific PV+ interneuron deficits.

## 1. Introduction

### 1.1. PV+ Interneuron Function in the Brain

Parvalbumin-positive (PV+) interneurons are a specific type of gamma-aminobutyric acid containing (GABAergic) inhibitory neuron found throughout the brain (see [1,2,3,4] for comprehensive reviews on different types of cortical, hippocampal, and cerebellar GABAergic neurons). GABAergic interneurons are essential in regulating the firing of the principal excitatory neurons in the central nervous system. They also play an important role in cortical sensory plasticity during development and adulthood [5,6]. Single-cell transcriptomics has revealed evolutionary conservation of regulatory networks during inhibitory interneuron development in humans and animals [7,8]. Clearly, proper functional connectivity and plasticity of neuronal networks within the developing and mature brain are dependent on a highly efficient and precisely coordinated GABAergic signaling system.

PV+ GABAergic interneurons comprise 30–50% of all inhibitory interneurons and represent the largest subpopulation of inhibitory cells in the cortex (for recent reviews see [9,10]). They express the calcium-binding protein parvalbumin. The other major inhibitory groups, based on the biochemical markers they express, are somatostatin (SOM) and vasointestinal peptide (VIP) interneurons [11]. These three classes of inhibitory interneurons are thought to account for 80–90% of all inhibitory neurons. The PV+ inhibitory interneurons are primarily fast spiking neurons that mostly target the somata and proximal dendrites of their target principal excitatory cells, and also other inhibitory cells including themselves [12,13,14,15,16,17,18]. They provide powerful but relatively short-lived inhibition [14,19,20,21,22], and play a vital role in feedforward and feedback synaptic inhibition, preventing run away excitation in neural networks [23,24,25,26,27]. They are also critical to the generation of gamma oscillations, a type of high-frequency neuronal oscillation linked to working memory and other cognitive processes including sensory perception and attention [28,29,30].

### 1.2. PV+ Interneuron Dysfunction in the Brain

Dysfunction of PV+ inhibitory interneurons has been implicated in various neurological disorders due to their crucial role in maintaining the balance between excitation and inhibition in the brain. Disruption of the normal excitatory/inhibitory (E/I) balance within specific neurocircuits in the brain has been linked to epilepsy [26,27,31], neurodevelopmental disorders [32], and neuropsychiatric conditions, including attention deficit hyperactivity disorder (ADHD), autism spectrum disorders, depression, anxiety, schizophrenia, bipolar disorder, and addiction [33,34,35,36,37]. Furthermore, dysfunctional inhibitory interneurons have been implicated in the pathogenesis of neurodegenerative diseases linked to aging [38]. Fast spiking PV+ GABAergic interneurons are particularly vulnerable to aging due to their high metabolic demands. Hence, PV+ interneuron deficits and degeneration have also been associated with cognitive decline and memory impairment in dementia and Alzheimer’s disease (AD). Interestingly, epileptic activity is frequently associated with AD, and seizures can hasten cognitive decline in AD patients [39]. One of the proposed mechanisms of epileptogenesis in AD is selective impairment of GABAergic interneurons in the hippocampus and parietal cortex [40].

The focus of the current review is on recent research into the role of dysfunctional PV+ inhibitory interneurons in the generation of epileptic seizures and cognitive impairments. The interrelationship between AD pathology and epileptic excitotoxicity is considered. New insights gleaned from recent cutting-edge techniques such as optogenetics, chemogenetics, and genetic manipulations are discussed. This review also examines the potential of targeting PV+ interneurons to alter brain physiology and counter brain dysfunction in disorders such as epilepsy and AD in future therapeutic approaches.

## 2. PV+ Interneuron Dysfunction in Epilepsy

Epilepsy comprises a broad spectrum of disorders involving unprovoked seizures. The generation of epileptic seizures is characterized by bursts of hypersynchronous firing of neurons leading to hyperexcitability within brain networks. The International League Against Epilepsy (ILAE) defines an epileptic seizure as: “*a transient occurrence of signs and/or symptoms due to abnormal excessive or synchronous neuronal activity in the brain*” [41,42,43]. For a diagnosis of epilepsy, an individual must have had at least two unprovoked seizures more than 24 h apart or one unprovoked seizure with an increased probability (≥60%) of having another seizure over the next 10 years [42,44]. Seizures are catgorised into focal onset, generalized onset, and unknown onset according to ILAE 2017 classification of epilepsies [42,45]. The term focal replaces the old terminology of ‘partial’ and refers to seizures that start in one area of the brain or in a group of cells in one side of the brain. Generalized seizures encompass both hemispheres of the brain, with groups of cells on each side of the brain affected simultaneously. Comorbidities are common in patients with epilepsy, and include neurological, neuropsychiatric, and neurobehavioral disorders (for recent reviews see [36,46,47,48]). The bidirectional relationship between epilepsy and comorbid psychiatric disorders has been well established through multiple animal and human studies [49]. Epilepsy is associated with a higher incidence of depression, anxiety, and suicidal thoughts. Conversely, patients with depression and anxiety are at increased likelihood of experiencing epilepsy later in life.

Impaired inhibition has been a consistent finding in human patients and animal models of epilepsy [50,51,52]. PV+ inhibitory interneuron dysfunction, in particular, can lead to increased susceptibility to seizures due to the crucial role they play in regulating the synchronization of neuronal firing critical for maintaining the balance between excitation and inhibition in the brain. Alterations in the number, morphology, and/or function of PV+ interneurons have been reported in different types of epilepsy [53]. Such changes can lead to a decrease in inhibitory tone and exacerbate hyperexcitability, contributing to the generation and propagation of epileptic activity. Therefore, it might seem intuitive and logical that an increase in the firing of inhibitory interneurons would effectively decrease network activity and thus have an overall anti-epileptic effect. However, research in human subjects and animal models with epilepsy indicates that the role of inhibitory interneurons in controlling network hyperexcitability is more complex than this [54,55,56]. In the following sections, the impact of PV+ inhibitory interneuron dysfunction in the generation of different types of epileptic seizures is considered in more detail, with an emphasis on the underlying cellular and molecular mechanisms that are causal rather than a consequence of altered brain function.

### 2.1. The Role of PV+ Interneuron Dysfunction in Absence-Seizures

Absence seizures are a type of generalized, nonconvulsive epileptic seizure, most common in children aged 3 to 14 years. In childhood absence epilepsy (CAE), seizures are identified by bilateral synchronous 3–4 Hz spike-wave discharges (SWDs) on the electroencephalogram (EEG), coincident with a lack of consciousness termed ‘absences’ [57]. Absence seizures are brief (a few seconds in duration) but frequent (up to hundred/day) episodes, during which the child is unaware, stops what he or she is doing, and appears to stare into space. Rodent models of CAE also display SWDs and concomitant behavioral arrest. Absence seizures are known to arise from altered E/I dynamics within the cortico-thalamocortical (CTC) network [58,59,60,61,62]. However, the underlying cellular and molecular mechanisms that cause the switch from normal physiological oscillations in the CTC network to pathological SWDs during absence seizures are not fully understood and appear to be multifactorial as evidenced by the different potential causative mechanisms identified in different rodent animal models, and the different responses to anti-epileptic drugs (AEDs) in humans [63]. Approximately one third of children with absence seizures are pharmaco-resistant.

In the stargazer mouse model, which exhibits SWD and behavioral arrest similar to humans, absence seizures are linked to impaired PV+ interneuron function within CTC microcircuits [64,65,66,67,68,69,70,71,72,73]. The CTC network comprises reciprocally connected glutamatergic relay neurons in the thalamus and pyramidal cells in the cortex, which allows for bidirectional communication between these two regions. Sensory information is relayed from the periphery through the ventroposterior (VP) thalamus to the somatosensory cortex via thalamocortical (TC) projections from the relay neurons. Pyramidal cells in the cortex send corticothalamic (CT) projections back to the relay neurons in the VP thalamus. PV+ interneurons are strategically positioned in the cortex to provide strong feedforward inhibition to pyramidal cells when activated by TC projections. In the thalamus, PV+ interneurons are located in a thin shell of tissue surrounding the VP nucleus called the reticular thalamic nucleus (RTN). They receive collaterals from both the CT and TC projections and provide feedforward inhibition to the relay neurons via the CT-RTN-VP pathway and feedback inhibition via TC-RTN-VP route (see [73] Fig.1). The CT-RTN-VP pathway is more dominant than the direct CT-VP route due to stronger synaptic excitation at CT-RTN connections [74]. The stronger CT-RTN synaptic strength allows feed-forward inhibition provided by PV+ interneurons to overcome direct cortical excitation of relay neurons. The thalamic relay neurons fire high-frequency bursts of action potentials when T-type calcium channels are activated. Generation of normal physiological oscillations within the CTC network arise via a sequence of feedforward inhibition from the RTN interneurons to VP relay neurons, leading to T-type calcium current dependent post-inhibitory rebound bursts of action potentials in the relay neurons, and then subsequent feedback inhibition via activation of RTN inhibitory interneurons by TC collaterals [75,76,77]. Thus, normal physiological oscillations within the CTC network require finely tuned firing of thalamic and cortical neurons within CTC microcircuits. Pathological oscillations, which appear as SWDs on the EEG, arise when the firing of these neurons is disrupted and the balance between excitation and inhibition is altered [58,59].

In the monogenic stargazer mouse model of absence epilepsy, firing of PV+ interneurons within CTC microcircuits is impaired due to a loss of α-amino-3-hydroxy-5-methyl-4-isoxazolepropionic acid receptors (AMPARs) at their input synapses. AMPARs mediate the majority of glutamatergic fast excitatory synaptic neurotransmission in the brain. The stargazer carries a mutation in the CACNG2 gene encoding a transmembrane AMPAR regulatory protein subunit (TARP γ2) also known as stargazin [78,79], which is responsible for trafficking AMPARs into synapses and modulating their function [80,81,82,83,84,85,86,87]. Stargazin is selectively expressed in PV+ interneurons in the cortex and thalamus [88,89]. Hence, there is a selective loss of AMPARs specifically at synaptic inputs onto PV+ interneurons in the somatosensory cortex [65,66,67] and at CT-RTN synapses in the thalamus [64,68], resulting in PV+ interneuron dysfunction and impaired feedforward inhibition within the CTC network. PV+ interneurons in RTN predominantly express AMPARs that contain the GluA4 subunit [90]. The *Gria4* knockout mouse, which lacks GluA4-AMPARs, also displays thalamic hyperexcitability, SWDs, and absence seizures [74]. Paz et al. [74] used optogenetics to dissect the cellular and microcircuit mechanisms underlying network hyperexcitability in *Gria4*^−/−^ mice. Channelrhodopsin-2 (ChR2) was virally injected into either the somatosensory cortex or the VP thalamus to allow selective optical stimulation of CT or TC axonal terminals, respectively, within the sensory thalamus. The strength of specific synaptic connections was measured by: (1) recording from individual relay neurons in VP while shining blue light on ChR2-labelled CT axonal terminals (CT-VP pathway); or (2) recording from RTN inhibitory interneurons while activating TC axonal terminals in animals where the VP thalamus was intact (VP-RTN); or (3) recording from RTN inhibitory interneurons after excision of the VP region and activating CT terminals (CT-RTN) (see [74] Figs. 4, 5, S2). These experiments demonstrated that absence of GluA4 (hence loss of GluA4-AMPARs) caused the strength of the CT-RTN synapse to be weakened and consequentially CT-RTN-VP feed-forward inhibition to be impaired. In contrast, both the CT-VP and VP-RTN pathways were unaffected. The direct CT-VP pathway thus became dominant, leading to hyperexcitation of relay neurons in VP. This then led to hyperexcitation of PV+ interneurons in RTN via the VP-RTN route, which allowed feedback inhibition of the relay neurons and thus subsequent post-inhibitory rebound bursts of action potentials; ultimately this sequence of events led to hypersynchronous pathological oscillations. Collectively, these data from the stargazer and *Gria4*^−/−^ models of absence epilepsy indicate that loss of feedforward inhibition from PV+ interneurons contributes to pathological SWDs and absence seizures in these mice.

However, while there is clear evidence from monogenic mouse models that impaired feedforward inhibition onto the principal excitatory neurons in the CTC network (cortical pyramidal cells and thalamic relay neurons) is one potential underlying mechanism for the generation of absence seizures, research from polygenic rat models indicates other cellular and molecular mechanisms can also contribute to the generation of SWDs. GABAergic inhibition can be phasic (mediated by synaptic GABA receptors) or tonic (mediated by extrasynaptic GABA receptors). Studies using the Genetic Absence Epilepsy Rat from Strasbourg (GAERS) have shown that increased tonic inhibition in the VP thalamus, mediated by extrasynaptic GABA_A_ receptors in relay neurons, is crucial in governing seizure genesis in this rodent model [91,92]. Enhanced tonic inhibition in the GAERS is due to compromised GABA uptake by a malfunction in the astrocytic GABA transporter GAT-1 leading to constitutively higher GABA levels in the thalamus, but not somatosensory cortex (for review see [62]). Maheshwari and Noebels [60], in their review of monogenic models of absence epilepsy, suggested a framework involving fast feedforward disinhibition as one common mechanism that could lead to increased tonic inhibition in the cortex and/or thalamus. Under this scenario, enhanced tonic inhibition causes hyperpolarization in the principal cells, which deinactivates T-type calcium channels and leads to reciprocal burst firing within the thalamocortical loop.

Direct evidence that loss of feedforward inhibition from functionally impaired PV+ interneurons can induce SWD and absence seizures has been provided by the use of Designer Receptor Exclusively Activated by Designer Drug (DREADD) technology [93,94]. Focal silencing of PV+ interneurons within cortical or thalamic feedforward microcircuits in non-epileptic mice using inhibitory Gi-DREADDs induced SWD-like oscillations on the EEG, which were associated with behavioral arrest and absence seizures [93]. Furthermore, selectively activating feedforward PV+ neurons (using excitatory Gq-DREADDs), following pentylenetetrazol (PTZ) induced seizures, either prevented SWDs and absence seizures or suppressed their severity [94]. Importantly, other studies [95,96,97,98] have shown that transplantation of interneuron precursor cells derived from the medial ganglionic eminence (MGE) into the cortex of young and adult mice reduces or abolishes seizures in genetic mice models of epilepsy; GABAergic interneurons originate in the MGE. Collectively, these studies provide evidence that feedforward disinhibition in CTC microcircuits (through dysfunctional PV+ interneurons) is one causative mechanism for the generation of SWDs and absence seizures.

### 2.2. The Role of PV+ Interneuron Dysfunction in Other Types of Epileptic Seizures

Disruption of the development and/or function of PV+ interneurons has also been linked to defective inhibitory GABAergic neural circuits underlying the pathogenesis of several other types of epilepsy. The most common type of focal onset seizure in humans is temporal lobe epilepsy (TLE), which affects approximately fifty million people globally. TLE is usually diagnosed in the first two decades of life (see [99] for a recent review of the epidemiology of TLE, and its pathophysiology, diagnosis, and management). TLE is associated with malfunctioning of the hippocampus years after an initial insult due to changes in GABAergic interneuron circuits [100,101,102]. Common risk factors for developing TLE later in life are traumatic brain injuries during birth or infections such as encephalitis or meningitis during childhood. Prolonged febrile seizures are also a common risk factor for TLE. Most patients with TLE have pronounced loss of neurons in limbic brain regions, including the hippocampus, on autopsy. The neurodegeneration in the hippocampus is known as hippocampal sclerosis and affects up to 70% of TLE patients.

PV+ interneurons are selectively lost in the hippocampus/subiculum region in both human patients and animal models of TLE [103,104,105]. Recently, Drexel et al. [102] demonstrated that sustained inhibition of GABA release from PV+ interneurons in the hippocampal CA1/subiculum region was sufficient to induce hyperexcitability and spontaneous recurrent seizures (SRS) in mice. They used tetanus toxin (virally delivered) to selectively and permanently inhibit GABA release from PV+ interneurons without destroying them. Hence, mice developed epilepsy without signs of neurodegeneration, mimicking non-lesional TLE in patients. Drexel et al. [102] also tested the impact of transient inhibition of GABA release from PV+ interneurons using DREADD technology. They selectively expressed the inhibitory designer receptor hM4Di in PV+ neurons to silence them for less than two hours. While this reduced the seizure threshold, it did not induce acute or recurrent seizures. Overall, the study by Drexel et al. [102] highlights the importance of inhibition mediated by PV+ interneurons in the hippocampus/subiculum and identifies the chronic loss of GABA release from dysfunctional PV+ interneurons as a potential mechanism in the development of TLE. In another study [106], GABAergic interneurons in the hippocampus were selectively ablated to test if focal interneuron lesions cause acute seizures as in status epilepticus (SE) and/or chronic epilepsy (i.e., persistent SRS). In this study, Gad2-ires-Cre knock-in mice (which have Cre recombinase expression directed to GAD2 positive neurons), were injected in the CA1 region with an adeno-associated virus containing the diphtheria toxin (DT) receptor. DT was subsequently injected to induce a focal ablation of GABAergic interneurons specifically in this brain region. All mice with DT-induced interneuron lesions had SRS but not SE; selective interneuron ablation rarely led to persistent SRS (i.e., epilepsy). Collectively, these studies [102,106] provide strong evidence that focal ablation or inactivation of GABAergic interneurons can lead to transient SRS in adult mice. However, the reported lack of persistent SRS in the Spampanato and Dudek study [106] could have been because recordings from interneuron-ablated mice were only conducted for a period ranging from 17–73 days (average of 35 days) after treatment. Other mouse studies have shown that SRS can occur months after the initial brain injury; in humans there can be latent periods of up to several years before the development of epilepsy. Hence, caution needs to be applied when interpreting data regarding the persistence of SRS based on a relatively short seizure recording period, which may not have been long enough to detect latent SRS as evidence for epileptogenesis. Interestingly, chemogenetic activation of hippocampal PV+ interneurons (transgenetically transduced with the excitatory DREADD muscarinic receptor hM3Dq) attenuated seizures in vivo in acute and chronic rodent models of TLE [107]. Overall, these data provide evidence that PV+ interneurons within the hippocampus could be a potential target for the treatment of TLE.

### 2.3. Conclusions on the Role of PV+ Interneuron Dysfunction in Epilepsy

The role of dysfunctional GABAergic signaling in the generation, regulation, and progression of epileptic seizures is complex. Studies from human patients and rodent models indicate multiple different mechanisms that may contribute to the initiation and progression of generalised and focal seizures in different brain regions. Some researchers [106] have posited a ‘two-hit model’ whereby more than one mechanism may be needed to induce progressive epilepsy in TLE e.g., certain genetic susceptibilities may be required apart from loss of GABAergic inhibition. Also, different mechanisms may come into play at different stages of disease pathogenesis. Nevertheless, from the preceding discussion of recent research into the cellular and molecular mechanisms underlying CAE (an example of a genetic, generalized onset epilepsy) and TLE (the most common type of focal epilepsy) there is compelling evidence for dysfunctional GABAergic signaling, and specifically impaired PV+ interneuron function, in the initiation and generation of seizures in CAE and TLE. Interestingly, mutation of the SCN1A gene, which encodes the α-subunit of the neuronal sodium channel NaV1.1 that is concentrated in the initial axon segment of PV+ GABA interneurons, reduces their excitability [108]. SCN1A is one of the most common epilepsy genes in humans. SCN1A mutant mouse models exhibit epileptic seizures due to reduced excitation of PV + interneurons and resulting hyperexcitation in several brain regions [109]. Collectively, these data provide evidence that PV+ interneuron dysfunction plays a central role in the pathogenesis of epilepsy. Furthermore, the recent application of DREADD and optogenetic technology to selectively manipulate PV+ interneuron function in vitro and in vivo to control seizure activity in various animal models of epilepsy [93,94,110,111,112,113,114], provides optimism that precision targeting of specific PV+ interneurons may offer future treatment strategies for epileptic patients with genetic mutations affecting PV+ neuron development and function.

## 3. PV+ Interneuron Dysfunction in Alzheimer’s Disease (AD)

While many genetic risk factors and early biomarkers have been identified for AD, the primary causal factor(s) responsible for initiating the cognitive decline in patients with AD remain highly controversial. Although toxic plaques and tangles correlate with progressive neuropathology, the ‘Amyloid Cascade Hypothesis’ [115,116], which proposes that deposition of the amyloid-β (Aβ) peptide in the brain is the primary underlying cause for the symptoms and progressive cognitive impairment in AD, has been hotly debated [117,118,119]. This has mainly been due to the repeated failure of clinical trials targeting Aβ over the past three decades, and also the extremely modest cognitive benefits (which are also highly controversial) achieved by recent monoclonal anti-amyloid drugs such as Aducanumab, Lecanemab, and Donanemab [120,121,122,123,124]. Furthermore, renowned longitudinal studies (e.g., The Nuns Study [125] and The +90 study [126]) have also demonstrated a lack of correlation between amyloid plaque formation in the brains of some aged patients on post-mortem and their known cognitive status prior to death [125,126,127,128].

PV+ inhibitory interneuron dysfunction has now been identified as one of the earliest pathophysiological perturbations in AD [129]. Altered E/I balance and impaired GABAergic signaling are known risk factors for AD [130,131]. Disruptions in circuit activity emerge before Aβ deposition in AD animal models and human patients. Furthermore, clinical evidence reveals an elevated comorbidity of epilepsy with AD [39]. Alzheimer’s patients are known to experience significantly more epileptic seizures than age-matched controls [40,132], and the prevalence of epilepsy prior to cognitive decline in AD sufferers has been reported to be over seventeen times higher than reference populations [133]. The reported incidence of epileptic seizures in AD patients varies depending on how the data have been collected; in closely monitored cohorts, rates as high as 64% have been found, whereas large scale epidemiological studies indicate that unprovoked seizures occur in 10–22% of patients with AD. Late-onset epilepsy and epileptiform activity can precede cognitive deterioration in AD by several years. Adult-onset epilepsy has even been proposed as a potential risk factor for later dementia and its presence has been shown to predict a faster disease progression (see [134] for a recent review of epilepsy in late-onset AD). 

### 3.1. PV+ Interneuron Dysfunction as a Key Pathogenic Mechanism in AD

Evidence specifically implicating PV+ interneuron dysfunction as a key pathogenic mechanism in AD-associated memory impairment has been provided from several animal and human studies. PV+ interneurons are responsible for coordinating the hippocampal network dynamics required for memory consolidation [135]. PV+ GABAergic interneuron dysfunction has been linked to altered network oscillations in the hippocampus and medial prefrontal cortex of a transgenic AD mouse (AppNL-G-F/NL-G-F), which models early stages of Aβ-induced network impairments [136]. Altered gamma and theta oscillations have also been associated with decreased numbers of hippocampal GABAergic neurons in the TgCRND8 mouse model of AD [137], which expresses a double-mutant form of the human APP gene. Alteration in brain network oscillations is a common finding in transgenic mouse models of AD; altered gamma and theta oscillations and associated cognitive deficits have been linked to selective impairment of GABAergic interneurons in the hippocampus and parietal cortex of several AD mice [138,139,140]. Additionally, slow-wave activity is severely altered in the hippocampus, neocortex, and thalamus of some AD models [141]. Slow-wave oscillations occur during sleep and are important for integration of information across brain regions involved in memory consolidation. In APP23xPS45 AD mice (double transgenic mice expressing a mouse/human amyloid precursor protein and a mutant human presenilin 1), amyloid-β impairment of slow-wave propagation and long-range circuit dysfunction can be rescued by increasing GABAergic transmission and restoring E/I balance [141]. Hence, slow wave sleep has been proposed as a promising intervention target for AD by some researchers [142].

Overall, the evidence from animal and human studies indicates that PV+ inhibitory interneuron deficits and altered network activity are linked to cognitive impairments in AD [138,143,144]. Moreover, early restoration of PV+ interneuron activity can prevent memory loss and network hyperexcitability in some mouse models of AD [145]. However, whether PV+ interneuron dysfunction is directly linked to causation, rather than a down-stream consequence of AD pathogenesis, is still open to debate (see reviews [38,146,147]).

### 3.2. Molecular Mechanisms Underlying PV+ Interneuron Dysfunction in AD

Evidence from animal studies indicates that potentially different types of PV+ interneuron dysfunction contribute to memory impairment at different stages of AD. Hypofunction of hippocampal PV+ interneurons has been reported in APP/PS1 mice at late stages of AD (i.e., at 7+ months in these mice). However, at three months of age, when memory deficits are first evident in APP/PS1 mice, hippocampal PV+ interneurons are transiently hyperexcitable, suggesting a biphasic mechanism [38,145].

Recently, Olah et al. [148] reported a novel mechanism underlying PV+ interneuron hypoexcitability in a mouse model of familial AD that rapidly develops severe amyloid pathology (i.e. the 5xFAD mouse, which expresses human APP and PSEN1 transgenes with a total of five AD-linked mutations). The hypoexcitability was found to be due to impairments in the biophysical properties of Kv3 potassium channels, which are known to underlie the ability of some neurons to fire action potentials at very high frequencies for short periods of time. PV+ fast-spiking GABAergic interneurons specifically express Kv3.1 and Kv3.2. Olah et al. demonstrated that dysregulation of Kv3 channel biophysics leads to impaired action potential generation in PV+ interneurons and dysregulation of cortical excitability in the 5xFAD mouse model. They proposed targeting alterations in biophysical ion channels as a potential strategy in the design of future therapies to ameliorate cortical circuit hyperexcitability in early AD. Human genetic studies have identified mutations in three out of the four subtypes of Kv3 channels as causal mutations linking Kv3 channel dysfunction to brain disorders [149]. Andrade-Talavera et al. [150] were able to modulate Kv3.1 and Kv3.2 electrophysiologically to rescue Aβ-induced desynchronization of fast-spiking interneuron firing in an in vitro AD model and thus restore gamma oscillations. Gamma oscillations are degraded in AD patients exhibiting cognitive impairment, with the degree of cognitive decline correlating with the severity of gamma disruption.

There is now a growing list of human genetic mutations linking Kv3 channel dysfunction in GABAergic interneurons to a range of debilitating disorders, including AD and epilepsy. Clatot et al. [151] used computational modelling of Kv3.2-expressing fast-spiking PV+ GABAergic interneurons to demonstrate how the Kv3.2-Cys125Tyr variant impairs neuronal excitability and dysregulates inhibition in cerebral cortex circuits resulting in epileptic seizures. In another study by Yeap et al., [152], reduction of potassium channel Kv3.4 levels was shown to ameliorate synapse loss in a mouse model of AD.

### 3.3. PV+ Interneuron Degeneration in AD

PV+ interneurons are known to degenerate early in AD pathogenesis [146,153]. This has been proposed to be related to the increased energy demands required for their high firing frequencies [15,18]. PV+ GABAergic dysfunction can promote downstream changes in Aβ and Tau pathologies associated with AD. However, the reverse is also true; an increase in Aβ and Tau toxicity can exacerbate hyperexcitability and PV+ neuronal degeneration. For example, toxic accumulation of Aβ peptides triggers synaptic degeneration, circuit remodeling, and abnormal synchronization within the hippocampal networks during human TLE [39]. One mechanism that has been proposed to account for the interrelationship between AD pathology and epileptic excitotoxicity in AD mouse models is an increase in the neurogenesis of new immature neurons within the dentate gyrus at early stages of the disease [154,155,156]. These immature cells are less likely to mature into GABAergic neurons leading to impairments of GABA transmission [157,158]. Transplantation of GABAergic interneuron progenitor cells into the hippocampus of APP/PS1 transgenic mice has been shown to rescue impaired synaptic plasticity and cognitive deficits along with a suppression of neural hyperexcitability [159].

### 3.4. Parvalbumin and Proteins Involved in Long-Term Plasticity

Several studies have shown that long-term plasticity can be monitored through altered levels of PV protein as well as activity-regulated-cytoskeletal-associated protein (Arc/Arg3.1). For example, long-term plasticity changes following auditory deprivation can be monitored through altered levels of Arc and PV protein in the auditory cortex and hippocampus, which can be correlated with changes in long-term potentiation (LTP) in hippocampal CA1 pyramidal neurons [160].

Hippocampal PV+ interneurons have been shown to play a critical role in memory development and the maturation of the hippocampus-dependent memory system [161]. Hippocampi show differential kinetic profiles of protein expression following learning at different stages of development [162]. Rodent studies have shown that learning during infancy is associated with significant increases in the levels of PV protein in the hippocampal CA1 region. Other studies in humans, monkeys, and rats have demonstrated that PV+ interneurons are also centrally involved in driving neurocognitive maturation during critical developmental periods. The levels of expression of both PV-mRNA and PV protein are significantly increased from childhood to adulthood in the prefrontal cortex, which plays a critical role in higher order cognitive aspects of memory processing and memory consolidation (for review see [163]).

In the brain, the expression of immediate-early genes (IEGs) has been widely used as a molecular marker for neuronal populations that undergo plastic changes underlying formation of long-term memory (for review see [164]). During early postnatal development, sensory regions of the brain undergo critical periods of heightened plasticity, which are important for learning and memory and in shaping adult neural networks for sensory perception [165]. Three IEG products and biomarkers of neuronal activation, namely c-FOS, Arc/Arg3.1, and ZIF268 (also known as EGR1 or NGFI-A), are expressed at significantly higher levels in the dorsal hippocampus and the medial prefrontal cortex at postnatal day 17 (PN17) compared with PN24 (juvenile age) and PN80 (young adult age) in rats [166,167]. Gao et al. [165] demonstrated that conditional removal of Arc/Arg3.1 during the first postnatal month, which represents a critical period when this IEG is transiently up-regulated in the hippocampus, alters oscillations in this brain region and diminishes spatial learning capacity throughout adulthood. In the adult brain, mRNA and proteins for IEGs have low basal expression levels but rapidly and transiently increase in response to stimulations that evoke LTP or long-term depression (LTD).

The expression of Arc/Arg3.1 protein also changes drastically during epileptic seizures. Arc protein is abundantly generated by neurons during epileptic seizures and affects epileptic susceptibility in rodent models. For example, Arc expression is upregulated in synapses of recently activated neurons of the epileptic seizure focal zone in TLE [168]. Interestingly, impaired Arc protein synthesis is also associated with other brain disorders, which have high mutual comorbidity with epilepsy, including memory disorders, AD, autism spectrum disorders, and schizophrenia (for a recent review see [168]).

While Arc/Arg3.1 is known to play a critical role in long-term synaptic plasticity, the mechanisms underlying the bidirectional regulation of synaptic strength are still under investigation. One proposed model is that Arc controls synaptic strength by regulating AMPAR trafficking and actin cytoskeletal dynamics in dendritic spines [169]. The IEG Narp (neuronal activity–regulated pentraxin) encodes a secreted synaptic protein that can bind to and induce clustering of AMPARs [170]. Narp is accumulated at excitatory synapses on PV+ interneurons where it regulates homeostatic scaling. Activity-dependent changes in the strength of excitatory inputs on PV+ interneurons have been shown to be dependent on Narp in acute hippocampal slices. *Narp−/−* mice have increased sensitivity to kindling-induced seizures. Chang et al. [170] proposed that Narp recruits AMPARs at excitatory synapses onto PV+ interneurons to restore the network E/I balance following episodes of increased circuit activity. Interestingly, rat hippocampus has significantly higher levels of AMPAR phosphorylation at the critical period PN17. Activity-dependent phosphorylation of AMPARs is important for regulating the delivery, stabilization and function of AMPARs at synapses, and thus for long-term plasticity. More recently, underlying molecular mechanisms have been identified, explaining how the CLOCK (circadian locomotor output cycles kaput) gene protein in inhibitory interneurons participates in neuronal activity and regulates the predisposition to epilepsy [171]. Clock genes control rhythms in physiology and behavior. Deng et al. (2024) showed that conditional knockout of the Clock gene in inhibitory neurons in a GAD-Cre;Clockflox/flox mouse caused upregulation of the basal protein level of Arc. Disruption of Clock in excitatory neurons caused alterations in cortical circuits, leading to the generation of focal epilepsy. Conversely, conditional Clock gene knockout in inhibitory neurons (in the GAD-Cre;Clockflox/flox mouse line), resulted in prolonged seizure latency, significant reduction in the severity and mortality of pilocarpine-induced seizures, and improved memory [171].

Collectively, these studies indicate that there is a correlation between activity-altered levels of PV protein and Arc protein expression in brain disorders characterised by seizures and cognitive deficits including epilepsy and AD. The Arc gene is associated with the pathogenesis of epilepsy; conversely, synaptic Arc protein synthesis is affected by seizures. Epileptic activation of Arc-mediated changes during seizures may affect memory consolidation in epilepsy and comorbid AD. The information from these studies may be important for developing future therapeutic strategies.

## 4. Targeting PV+ Interneurons in Future Treatment Strategies for Alzheimer’s Disease and Epilepsy

Various studies suggest inhibitory interneurons, especially PV+ interneurons, are susceptible to mitochondrial impairment. Proteomic analysis using cell-type-specific in vivo biotinylation of proteins (CIBOP) coupled with mass spectrometry to obtain native-state proteomes of PV interneurons has revealed strong correlations between PV+ interneuron-specific proteome signatures and progressive neuropathology in humans and mouse models of Aβ pathology [129]. Analysis revealed unique signatures of increased mitochondrial and metabolic proteins in response to early Aβ pathology. Recently, Olkhova et al. [172] developed a mouse model of mitochondrial dysfunction in PV+ interneurons that has mitochondrial and cognitive defects resembling those observed in patients. This mouse model has the potential to be used as a drug screening platform towards discovery of future therapeutics to treat severe neurological impairment arising from mitochondrial dysfunction in PV+ interneurons.

Other studies have demonstrated that the destruction of PV+ interneurons, mediated by microglia, plays a key role in cognitive impairments associated with systemic inflammation [173]. Interestingly, a recent investigation into the mechanisms underlying the beneficial effects of regular exercise for improving learning and memory functions across multiple neurological diseases has revealed that engaging in exercise protected parvalbumin interneurons, in a mouse model of epilepsy, via the suppression of neuroinflammation [174].

Glial progenitor cells have been suggested as a suitable target for future therapeutic intervention strategies aimed at generating new GABAergic interneurons via in situ reprogramming [163]. Until recently, reprogramming of human glial cells remained challenging despite the successful conversion of mouse glia into interneurons. Giacomoni et al. [175] were able to successfully convert human stem cell-derived glial progenitor cells (hGPCs) into functional GABAergic interneurons, within a month. The induced GABAergic neurons exhibited complex neuronal morphologies with extensive dendritic trees and expressed subtype-specific interneuron markers (e.g., parvalbumin). The authors proposed that these induced interneurons could be of potential use for development of therapies for interneuron pathologies implicated in several neurological disorders.

Finally, the development of optogenetics [110,111,112,113,114,176,177] and chemogenetic techniques such as DREADDs [178,179] and their recent successful application, in vitro and in vivo, to attenuate some types of epileptic seizures arising from PV+ interneuron dysfunction in rodent models [93,94,180], holds out promise that these technologies may be able to be used in the future to treat neurological disorders in humans arising from disruption in the development or function of PV+ GABAergic interneurons.

## 5. Conclusions

In conclusion, there is now compelling evidence, from in vitro and in vivo studies in animal models and from human clinical and post-mortem data, that dysfunctional PV+ GABAergic interneurons play a central, pivotal role in neurological disorders such as epilepsy and AD. Developmental and functional deficits in PV+ inhibitory interneurons, leading to E/I imbalance within brain networks, may explain the interrelationship between epileptic seizures and cognitive impairments in AD. The comorbidity between AD and epilepsy is now well known. Defective PV+ interneuron function may represent one underlying mechanism linking epileptic excitotoxicity and cognitive decline in AD pathology. PV+ interneurons therefore offer potential targets for future therapeutic approaches in the treatment of epilepsy and AD.

Optogenetic and chemogenetic technologies such as DREADD, which allow specific populations of neurons to be precisely controlled (temporally and spatially), could be used in targeted treatment strategies where selective control of PV+ interneurons firing has been identified as an option in the treatment of patients with seizures and cognitive deficits. Further research will need to be undertaken to clearly differentiate causative mechanisms from downstream consequential changes and to identify which specific population of interneurons in the brain to target and when to manipulate them for optimum therapeutic benefits [181,182]. The specific underlying cellular and molecular alterations directly responsible for seizure generation and cognitive deficits in epilepsy and AD are still under study and are highly contentious. Nevertheless, from recent emerging data, PV+ interneurons are highly attractive candidates to target in the development of future treatments [183] for a range of neurological disorders involving seizures and cognitive decline. Further research into the specific mechanisms underlying PV+ interneuron dysfunction in neurological disorders will undoubtedly lead to the development of more precisely targeted therapeutic interventions for treating epilepsy and AD in the future.
